# Early-life adversity predicting the incidence of multisite chronic pain in the general population

**DOI:** 10.1192/j.eurpsy.2024.1753

**Published:** 2024-10-08

**Authors:** Isabelle Rouch, Marie-Pierre F. Strippoli, Jean-Michel Dorey, Bernard Laurent, Setareh Ranjbar, Pedro-Manuel Marques-Vidal, Chantal Berna, Marc Suter, Julien Vaucher, Armin von Gunten, Martin Preisig

**Affiliations:** 1 Memory Clinical and Research Center of Saint Etienne (CMRR) Neurology Unit, University Hospital of Saint Etienne, Saint Etienne, France; 2 INSERM, U1219, ACTIVE Team, Bordeaux Population Health Center, University of Bordeaux, Bordeaux, France; 3 Department of Psychiatry, Psychiatric Epidemiology and Psychopathology Research Center, Lausanne University Hospital and University of Lausanne, Prilly, Switzerland; 4 Department of Aging Psychiatry, Hospital Le Vinatier, Bron, France; 5 Service of Old Age Psychiatry (SUPAA), Department of Psychiatry, Lausanne University Hospital and University of Lausanne, Prilly, Switzerland; 6 INSERM, U1028; CNRS, UMR5292; Neuropain Team, Lyon Neuroscience Research Center, Lyon, France; 7 Department of Internal Medicine, Lausanne University Hospital and University of Lausanne, Lausanne, Switzerland; 8 Center for Complementary and Integrative Medicine, Department of Anesthesiology, Lausanne University Hospital and University of Lausanne, Lausanne, Switzerland

**Keywords:** Adverse childhood events, cohort, chronic pain, longitudinal

## Abstract

**Introduction:**

Adverse childhood events (ACEs) have been linked to widespread chronic pain (CP) in various cross-sectional studies, mainly in clinical populations. However, the independent role of different ACEs on the development of different types of CP remains elusive. Accordingly, we aimed to prospectively assess the associations between specific types of ACEs with the development of multisite CP in a large population-based cohort.

**Methods:**

Data stemmed from the three first follow-up evaluations of CoLaus|PsyCoLaus, a prospective population-based cohort study of initially 6734 participants (age range: 35–75 years). The present sample included 1537 participants with 2161 analyzable intervals (49.7% men, mean age 57.3 years). Diagnostic criteria for ACEs were elicited using semi-structured interviews and CP was assessed by self-rating questionnaires. Multinomial logistic regressions with generalized estimating equations method analyzed the relationship between the different ACEs measured in the beginning of the interval and the risk of developing multisite CP during the follow-up. Sensitivity analyses were performed to assess the predictive value of ACEs on multisite CP with neuropathic features.

**Results:**

Participants with a history of parental divorce or separation had an increased risk of developing multisite CP at during follow-up in comparison to those without (RR1.98; 95% CI 1.13–3.47). A strong association was highlighted between parental divorce or separation and the risk of subsequent CP with neuropathic characteristics (RR 4.21, 95% CI 1.45–12.18).

**Conclusion:**

These results highlight the importance of psychotherapeutic management of people experiencing parental separation to prevent CP in the future.

## Introduction

Chronic pain (CP) is defined as pain lasting more than 3 months; its prevalence varies from 17 to 40% in the general adult population and increases with age [1, 2]. CP remains a major health problem, impacting patients’ self-perceived state of health, severely affecting the quality of their social and professional lives, and closely linked to depressive symptoms [3]. CP mean duration is widely varying according to its etiologies, and in a previous paper, we found that 25% of CP suffers living in the community recovered from their pain over 5 years [4]. Independently from comorbidities, CP is associated with low educational level, socioeconomic status, female gender, and mood disorders impacting on patient-perceived health status, their quality of social and working lives, with a significant link to depressive symptoms [5, 6]. In middle-aged and older community-based adults, CP is mainly related to rheumatologic conditions. However, even in this population, CP mechanism may remain unexplained, as in the case of nociplastic pain without objective lesions [7]. The main pathophysiological mechanism for developing nociplastic pain is central sensitization in which pain amplification and hypersensitivity occur. Nociplastic pain may be defined by multisite localization and some painful characteristics found by DN4-7 scale designed to assess neuropathic pain but showing an overlap with nociplastic one [8]. Research has shown that adverse childhood events (ACEs) have lifelong effects on mental health, health-damaging behaviors, relational functioning, and physical health [9, 10], including CP. ACEs promote central pain sensitization by modifying cerebral plasticity in early life [11]. Few studies assessed prospectively the link between ACEs and the subsequent development of CP [12].

People experiencing this type of CP reliably show higher rates of adverse life experience [12] compared with the general population, and these biopsychosocial factors predict onset, quality, chronicity, and severity of pain [13–15]. Specifically, these ACEs and traumatic exposures have been associated with a greater prevalence of multisite CP, particularly nociplastic pain [16–18].

Most previous research on the associations between adverse life experiences and traumatic exposures with CP was focused on specific clinical populations. Thus, ACEs were associated with chronic widespread pain and fibromyalgia in numerous cross-sectional studies [16, 19–23]. However, the association between ACEs and CP may reflect selection and recall bias in cross-sectional studies [22] inciting to explore longitudinal data. A large-scale prospective cohort in the United Kingdom reported an association between several adverse events collected during childhood and the presence of CP in adulthood, independently from psychological distress [24]. In most studies, the analyses focused on the relationship between physical or sexual abuse and CP. However, the role of other types of ACEs and their impact according to age of onset on the later development of different types of CP is still debated. Some previous studies have highlighted an increased risk of developing CP in adulthood [17], but the respective roles of adversity, personality and mood disorders in the development of CP remain unclear. Numerous studies have demonstrated the link between ACEs and functional CP in children, adolescents, and young adults, particularly in clinical populations [25]. On the other hand, the origin of certain forms of adult-onset CP is not well understood; in particular, little is known about the potential link between ACEs and the onset of CP in this population.

In a previous study of a large population of middle-aged and older community dwellers initially free of CP, we showed that higher neuroticism and extraversion, and to a lesser extent the presence of ACEs, influenced CP occurrence independently from anxiety or major depressive disorders (MDD) [4]. Hence, we aimed to assess the prospective association between the number and different types of ACEs reported in the beginning of a follow-up (FU) interval and the subsequent development of multisite and non-multisite CP during this interval with and without subtyping into neuropathic versus non-neuropathic CP, controlling for various potential confounders, including demographic factors, personality traits, and lifetime major depressive and anxiety disorders.

## Methods

### Participants

**Figure 1.**
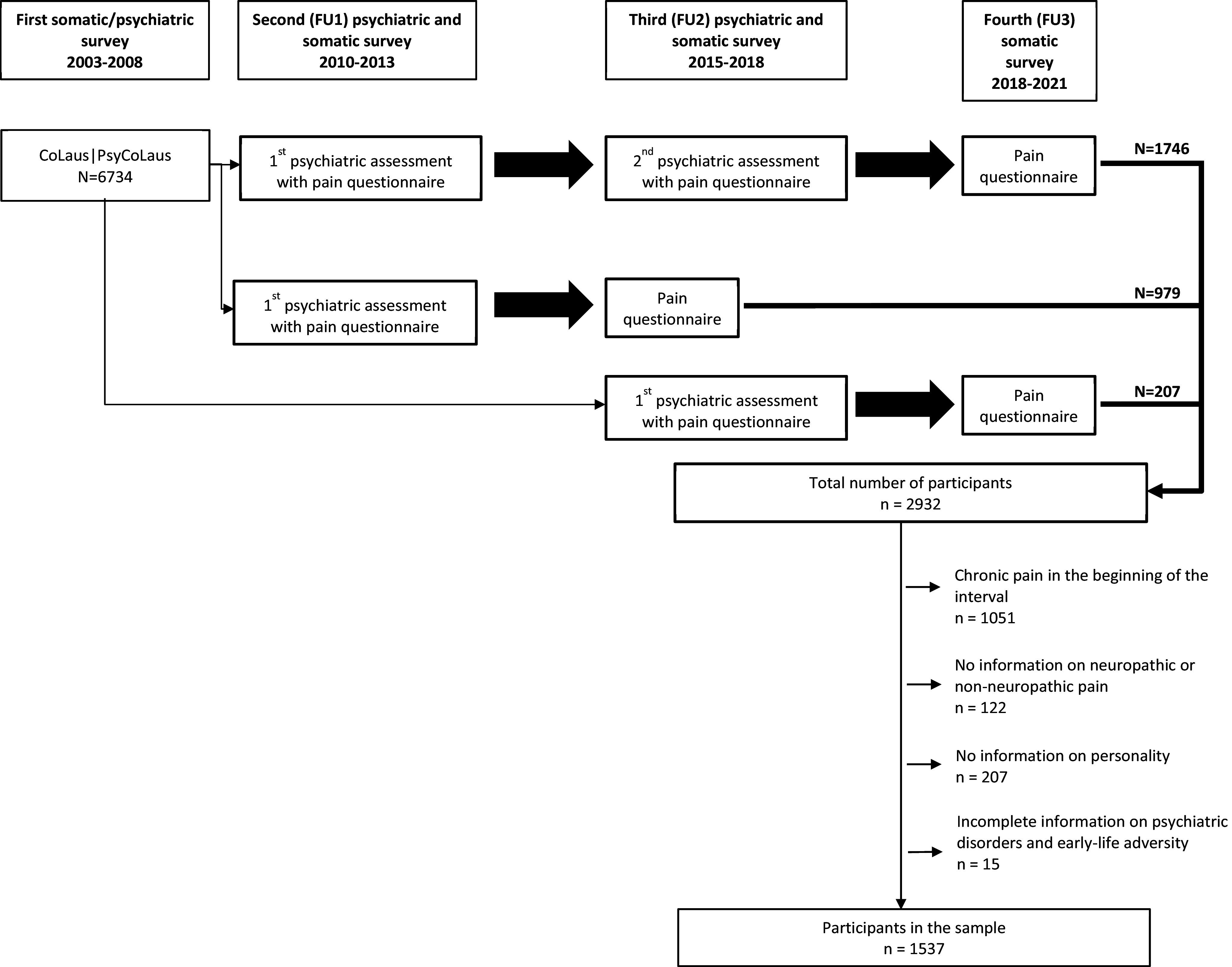
Flowchart of CoLaus|PsyCoLaus for the study of personality traits and early-life adversity and the incidence of multisite chronic pain. Analyzable interval.

The present data stem from CoLaus|PsyCoLaus, a prospective population-based cohort study designed to investigate cardiovascular risk factors and mental disorders in the community and to determine their associations. The study was previously described in detail [26, 27]. CoLaus|PsyCoLaus initially included a sample of 6734 participants (age range: 35–75 years) randomly selected from the residents of the city of Lausanne, Switzerland, between 2003 and 2006. After the first physical and psychiatric assessment, which took place between 2003 and 2008, the cohort was followed between 2009 and 2013 (FU1), 2014 and 2018 (FU2) as well as between 2018 and 2021 (FU3). Since FU FU1, participants have been invited to complete pain questionnaires during the physical investigation. The duration of the FU interval between FU1 and FU2 was 5.2 (s.d. 0.5) years and that between FU2 and FU3 was 3.8 (0.4) years. A total of 2932 participants completed both the psychiatric evaluation and the pain questionnaires at least at two of the three FU evaluations (Figure 1). After excluding individuals with CP in the beginning of the interval (an interval corresponds to a period between two pain assessments from FU1 to FU2 or from FU2 to FU3), missing information on personality, neuropathic or non-neuropathic pain, or type of ACEs, the final sample included 1537 participants with 2161 analyzable intervals (49.7% men, mean age [s.d.] 57.3 [9.7] years) consisting of 1215 and 946 analyzable intervals from FU1 to FU2 and from FU2 to FU3, respectively. For the 1746 participants who completed the psychiatric evaluations and the pain questionnaires at all three FU evaluations, the two intervals from FU1 to FU2 and from FU2 to FU3 were separately analyzed.

### Assessments

#### Pain

Participants underwent pain assessments at FU1, FU2, and FU3 using the STOPNEP questionnaire, an 11-question pain inventory designed and validated for epidemiological studies [2]. The first two questions aimed at identifying the presence of daily pain for at least three months, which define CP according to the International Association for Study of Pain [28].

Participants who responded positively to these two questions had to locate their pain from a list of body parts and to report the location of the most troublesome pain if appropriate. The number of pain sites was calculated for each participant, and according to literature [21] multisite CP was defined as follows: “multisite” when the number of pain sites was >3 sites, and “non-multisite” for one to three sites.

DN4 questionnaire (short form with seven questions) [29] was used to assess pain with neuropathic characteristics among participants with CP. The original version of the DN4 questionnaire consists of 10 items, in which 7 items are related to pain characteristics and 3 items are related to findings on physical examination of the painful areas. A short version based on the interview items was validated by the same authors for epidemiological studies. A score of 1 was given to each positive item and a score of 0 to each negative item. The total score was calculated as the sum of seven items. Participants with a total score ≥3 are considered to have neuropathic pain characteristics [2].

#### ACEs, mood and anxiety disorders, personality traits, sociodemographic characteristics, and other covariates

Diagnostic information on mental disorders was elicited at each psychiatric evaluation using the French version [30] of the DIGS [31]. Interviewers were master-level psychologists trained over at least a one-month period. An experienced senior psychologist reviewed all interviews and diagnostic assignments. The French version of this instrument has adequate inter-rater and test–retest reliability for major mood disorders [32]. The DIGS was completed with the post-traumatic stress disorder (PTSD) and the generalized anxiety disorder (GAD) sections of the French version [33] of the Schedule for Affective Disorders and Schizophrenia-Lifetime and Anxiety disorder version (SADS-LA) [34], and the brief phobia chapter of the DIGS was replaced by the corresponding more extensive chapters of the SADS-LA. In addition, a section on childhood events from the SADS-LA was incorporated in the DIGS. At the FU evaluations, a shortened version of the DIGS was used focusing on the period since the last assessment. Lifetime diagnoses of MDD and anxiety disorders (agoraphobia, panic disorder, GAD, social phobia) until the first pain assessment were assigned according to the Diagnostic and Statistical Manual of Mental Disorders-fourth version [35].

Information about ACEs occurring before the age of 17 years was elicited at the first psychiatric evaluation using the questions on traumatic events in the PTSD and the childhood sections of the SADS-LA. The following stressful experiences were considered as indicators of early-life adversity: sexual or physical abuse (PTSD section), witnessing of violence between parents, divorce or separation of parents, and loss of close relatives (parents or siblings) (childhood section) [36]. According to the suggestion by Friedman et al. [37], exposure to early-life adversity was also quantified by the sum of reported events.

Regarding personality traits, Neuroticism and Extraversion were assessed by the Eysenck Personality Questionnaire-Revised (EPQ-R) [38], which was completed at baseline and FU2, and the NEO Five-Factor Inventory (NEO-FFI-R) [39] completed at FU1. If participants had completed the two questionnaires prior to the beginning of the analyzed FU interval, data from the EPQ-R were used. NEO-FFI score was used for participants with missing data on EPQ-R. Neuroticism and Extraversion scores from the two questionnaires were normalized, and the z-scores were used for analyses.

During the physical evaluations, information was collected on sociodemographic characteristics including age, sex, education as well as medication (analgesics [ATC code N02], opioids [ATC code N02A + N02BG01], antidepressants [ATC code N06A], and anxiolytics [ATC code N05B]). Education was categorized into four levels: compulsory school, apprenticeship, high school/college, and university degrees.

#### Sleep and fatigue

Previous studies have shown an overlap between neuropathic symptoms diagnosed with the DN4-7 and fibromyalgia, defined by multisite musculoskeletal pain accompanied by fatigue and sleep disturbance. In order to better characterize the subtype of CP linked to ACEs (i.e., DN4+ multisite pain) and to determine whether these subjects suffered from fibromyalgia, the prevalence of sleep disorders and fatigue were assessed.

Sleep quality was assessed using Pittsburgh Sleep Quality Index (PSQI), a self-administered questionnaire assessing sleep quality and disorders for the month preceding the evaluation [40]. The participants were asked to rate each component using a 4-point Likert scale, ranging from 0 to 3 points with a maximum overall score of 21 points. A score >5 reflects sleep disorders.

Fatigue was measured by the Krupp fatigue severity scale (FSS), consisting of a nine-item questionnaire [41] and validated in the community in a Swiss population [42]. Respondents answered using a Likert scale ranging from 1 (completely disagree) to 7 (completely agree), a high score indicating a high level of fatigue. For each respondent, the sum scores for the nine items were divided by the number of items. A score >4 is considered as pathological.

### Statistical analysis

Participants who had no CP in the beginning of the interval were divided into three groups according to the CP status at the end of the interval: no CP, non-multisite (one to three sites) CP, multisite (>3 sites) CP. Continuous variables were presented as mean with standard deviation (s.d.) and categorical variables were expressed as percentages. To explore the association between early-life adversity (witnessing of violence between parents, divorce or separation of parents, loss of close relatives [parents or siblings], sexual and physical abuse, and number of childhood events) and the incidence of multisite CP at the end of the interval, namely no CP, non-multisite CP, multisite CP, multinomial logistic regression analyses were performed, as the proportional odds assumption was also rejected after score test for ordinal logistic. The multinomial logistic regression models were fitted with no CP at the end of the interval as the reference level. These models were all adjusted for sex; age; education; duration of FU; FU interval (FU1 to FU2 or FU2 to FU3); medication (analgesics, antidepressants, anxiolytics) at the end of the interval; presence of non-CP at the beginning of the interval; personality traits (neuroticism, extraversion); lifetime mental disorders (MDD and anxiety disorders); and intrapersonal correlations were taken into account by using generalized estimating equation methods. Model 1 was separately run for number of childhood events, witnessing of violence between parents, divorce or separation of parents, loss of close relatives (parents or siblings), and sexual and physical abuse. Model 2 included all types of ACEs simultaneously. Moreover, to better specify the psychological profile of the participants, the rate of MDD history in participants’ parents was compared between those with and without parental divorce or separation; finally, the age of the participants when their parents divorced was compared between the CP groups among participants with parental divorce.

To specify the type of CP may be influenced by early psychological factors, complementary analyses were conducted with regard to neuropathic pain characteristics assessed by DN4 scale. To explore the association between the different ACEs and the incidence of neuropathic or non-neuropathic multisite CP at the end of the interval, a five-category variable was created as follows: “no CP,” “non-multisite non-neuropathic (DN4−) CP, “non-multisite neuropathic (DN4+) CP,” “multisite non-neuropathic (DN4−) CP,” and “multisite neuropathic (DN4+) CP,” and multinomial logistic regression analyses were performed with no CP at the end of the interval as the reference level.

To better characterize DN4 characteristics, they were described in detail for subjects from “multisite DN4+ CP” group. The proportion of subjects with sleep disorders according to PSQI and fatigue according to FSS were compared between participants with “multisite DN4+ CP” and those without CP.

The results were considered as statistically significant at the level of p-value <0.05. All statistical analyses were conducted using SAS software (V. 9.4) (SAS Institute Inc., Cary, NC, USA).

## Results

### Descriptive results

#### Pain locations

Among subjects exhibiting CP, the most frequent locations concerned back (54.3%, 43.8% with lower back), knees (32.5%), shoulders (29.4%), feet (22.9%), hips (22.6%), and hands (20.5%).

The sociodemographic and clinical characteristics of the participants according to “multisite CP,” “non-multisite CP,” and “no CP” at the end of the interval are presented in Table 1.

**Table 1.** Description of the sample according to the presence of multisite chronic pain in the end of the follow-up interval among participants without chronic pain in the beginning of the follow-up interval

	All n = 2161	Multisite CP n = 89	Non-multisite CP n = 434	No CP n = 1638
Sociodemographic factors				
Age in the beginning (years), mean (s.d.)	57.3 (9.7)	58.1 (10.7)	58.0 (10.0)	57.1 (9.5)
Men, %	49.7	28.1	47.0	51.6
Education level, %				
University	28.2	21.4	25.8	29.2
High education except for university	28.7	25.8	28.8	28.9
Apprenticeship	34.0	33.7	37.6	33.0
Compulsory school	9.1	19.1	7.8	8.9
Length of follow–up (years), mean (s.d.)	4.6 (0.9)	4.7 (0.9)	4.7 (0.8)	4.6 (0.9)
Personality (z–score), mean (s.d.)				
Neuroticism	–0.19 (0.95)	0.30 (1.08)	–0.04 (0.93)	–0.25 (0.93)
Extraversion	0.02 (0.99)	0.04 (1.01)	0.06 (1.05)	0.01 (0.97)
Medication in the beginning, %				
Analgesics	1.9	5.6	2.5	1.5
Opioids	0.3	0.0	0.7	0.2
Antidepressants	5.9	9.0	9.0	4.9
Anxiolytics	2.3	6.7	2.3	2.0
Depression and anxiety in the beginning, %				
Lifetime major depressive disorder	43.1	60.7	50.5	40.2
Lifetime anxiety disorders (any)	17.8	21.3	19.8	17.0
Agoraphobia	3.1	4.5	4.4	2.7
Panic disorder	2.0	0.0	2.8	1.9
Generalized anxiety disorder	2.9	3.4	3.7	2.6
Social phobia	12.3	19.1	11.1	12.2
Sleep and fatigue in the beginning, mean (s.d.)				
Sleep (PSQI)	4.18 (2.65)	6.27 (3.25)	4.69 (2.77)	4.04 (2.59)
Fatigue (FSS)	2.67 (1.28)	3.24 (1.54)	2.77 (1.33)	2.63 (1.25)
Pain in the beginning, %				
Non chronic pain	10.0	24.7	16.8	7.4
Pain in the end^a^, %				
Neuropathic chronic pain	11.9	28.1	8.5	NA
Medication in the end, %				
Analgesics	4.5	19.1	7.1	3.0
Opioids	0.6	3.4	0.7	0.4
Antidepressants	6.2	13.5	9.7	4.8
Anxiolytics	2.5	6.7	2.8	2.2

Abbreviations: CP, chronic pain; s.d., standard deviation; NA: not available.

#### Prevalence of ACEs among the participants

Among the participants, exposure to at least one ACE was common, respectively, 38.2% of the subjects from “multisite CP” group, 28.8 % of the “non-multisite CP,” and 24.7% of the “no CP” one. Furthermore, 19.5% of the subjects were exposed to one traumatic event, 5.3% were exposed to two, and 1% were exposed to three such events.

### Crude associations between ACEs and subsequent multisite CP

The prospective associations between ACEs assessed in the beginning of the interval and multisite CP emerging during the FU interval are displayed in Table 2. The models run for the number of ACEs and each type of ACE separately (Model 1) revealed a higher risk of developing multisite CP in participants who had reported a higher number of ACEs, and in those who had been exposed to parental divorce or separation in comparison to individuals without these exposures. Moreover, participants who had lost a parent or a sibling had a greater risk of having “non-multisite CP” than those without CP. When all ACEs were assessed in a single model (Model 2), only the association of parental divorce or separation with a higher risk of developing multisite CP remained significant.

**Table 2.** Associations between early-life (before age 17 years) adversity and the presence of multisite chronic pain in the end of the follow-up interval among participants without chronic pain in the beginning of the follow-up interval (n = 2161)

					Chronic pain status in the end of the interval			
					Model 1		Model 2	
Early-life adversity (before age 17 years)	All n = 2161	Multisite CP n = 89	Non-multisite CP n = 434	No CP n = 1638	Multisite vs. no CP RR (95CI)	Non-multisite vs. no CP RR (95CI)	Multisite vs. no CP RR (95CI)	Non-multisite vs. no CP RR (95CI)
Number of early–life event or abuse, mean (s.d.)	0.34 (0.63)	0.57 (0.85)	0.37 (0.64)	0.31 (0.61)	1.27* (1.06;1.52)	1.07 (0.96;1.19)	–	–
Type of early–life event or abuse, %								
Sexual or physical abuse	63 (2.9)	5 (5.6)	12 (2.8)	44 (2.7)	1.19 (0.43;3.31)	0.78 (0.39;1.55)	0.99 (0.34;2.87)	0.78 (0.39;1.56)
Witnessing of violence between parents	236 (10.9)	15 (16.9)	51 (11.8)	170 (10.4)	1.37 (0.77;2.43)	1.07 (0.75;1.52)	1.08 (0.60;1.94)	1.04 (0.72;1.51)
Divorce or separation of parents	238 (11.0)	19 (21.3)	49 (11.3)	170 (10.4)	2.04* (1.18;3.53)	1.08 (0.76;1.53)	1.98* (1.13;3.47)	1.07 (0.74;1.54)
Loss of a close	190 (8.8)	11 (12.4)	48 (11.1)	133 (8.1)	1.52 (0.78;2.95)	1.43* (1.00;2.04)	1.52 (0.78;2.95)	1.41° (0.99;2.02)

*Note: °p* < 0.1, *p < 0.05.

Model 1: Each line corresponds to one multinomial logistic regression model.

Model 2: One single multinomial logistic regression model with each type of early-life adversity entered simultaneously.

All models were adjusted for sex; age; education; duration of follow-up interval; follow-up interval (FU1 to FU2 or FU2 to FU3); medication (analgesics, antidepressants, anxiolytics) in the end of the follow-up interval; presence of non-chronic pain at the beginning of the follow-up interval; personality (neuroticism, extraversion); lifetime mental disorders (major depressive disorder, anxiety disorders [agoraphobia, panic disorder, generalized anxiety disorder, social phobia]); and intrapersonal correlations.

The multinomial logistic regression models were fitted with no chronic pain in the end of the interval as the reference level.

Abbreviations: CP, chronic pain; RR, relative risk; 95CI, 95% confidence interval.

Bold entries mean significant results.

Age of exposure has been assessed for ACEs. No significant association was found between age of exposure to ACEs and risk of CP occurrence (data not shown).

### Relationship between ACEs and subsequent multisite CP with neuropathic characteristics

The association between ACEs and multisite CP with respect to neuropathic characteristics (DN4+ and DN4−) is presented in Table 3. Our results revealed a higher risk of developing multisite DN4+ CP in subjects with parental divorce or separation history when compared to participants without parental divorce or separation history.

**Table 3.** Associations between early-life (before age 17 years) adversity and the presence of neuropathic and non-neuropathic multisite chronic pain at the end of the follow-up interval among participants without chronic pain in the beginning of the follow-up interval (n = 2161)

						Chronic pain status at the end of the follow-up interval							
						Model 1				Model 2			
	Multisite CP		Non-multisite CP		No CP	Multisite		Non-multisite		Multisite		Non-multisite	
	DN4 + n = 25	DN4 - n = 64	DN4 + n = 37	DN4 - n = 397	n = 1638	DN4 + vs. no CP RR (95CI)	DN4 − vs. no CP RR (95CI)	DN4 + vs. no CP RR (95CI)	DN4 − vs. no CP RR (95CI)	DN4 + vs. no CP RR (95CI)	DN4 − vs. no CP RR (95CI)	DN4 + vs. no CP RR (95CI)	DN4 − vs. no CP RR (95CI)
Number of early–life event or abuse, mean (s.d.)	0.76 (0.93)	0.50 (0.82)	0.49 (0.69)	0.36 (0.64)	0.31 (0.61)	1.60** (1.21;2.13)	1.15 (0.92;1.43)	1.22 (0.93;1.58)	1.05 (0.94;1.18)	–	–	–	–
Type of early–life event or abuse (n, %)													
Sexual or physical abuse	2 (8.0)	3 (4.7)	3 (8.1)	9 (2.3)	115 (2.7)	1.78 (0.34;9.38)	0.94 (0.27;3.31)	2.15 (0.55;8.46)	0.64 (0.30;1.36)	0.93 (0.14;5.95)	0.95 (0.27;3.33)	1.90 (0.47;7.73)	0.66 (0.31;1.40)
Witnessing of violence between parents	6 (24.0)	9 (14.1)	6 (16.2)	45 (11.3)	170 (10.4)	2.50* (1.09;5.73)	1.05 (0.51;2.18)	1.48 (0.59;3.70)	1.03 (0.71;1.49)	1.60 (0.67;3.80)	0.95 (0.45;2.00)	1.18 (0.39;3.56)	1.03 (0.70;1.51)
Divorce or separation of parents	9 (36.0)	10 (15.6)	7 (18.9)	42 (10.6)	170 (10.4)	4.82** (1.88;12.32)	1.33 (0.68;2.62)	1.90 (0.80;4.52)	1.00 (0.69;1.45)	4.21** (1.45;12.18)	1.35 (0.69;2.63)	1.74 (0.66;4.56)	1.00 (0.68;1.47)
Loss of a close	1 (4.0)	10 (15.6)	2 (5.4)	46 (11.6)	133 (8.1)	0.49 (0.07;3.56)	1.91° (0.94;3.88)	0.72 (0.17;3.00)	1.50* (1.04;2.15)	0.52 (0.07;3.93)	1.90° (0.93;3.88)	0.71 (0.17;3.03)	1.48* (1.03;2.13)

*Note:* °*p* < 0.1; *p < 0.05; **p < 0.01.

Model 1: Each line corresponds to one multinomial logistic regression model.

Model 2: One single multinomial logistic regression model with each type of early-life adversity entered simultaneously.

All models were adjusted for sex; age; education; duration of follow-up; follow-up interval (FU1 to FU2 or FU2 to FU3); medication (analgesics, antidepressants, anxiolytics) at the end of the follow-up interval; presence of non-chronic pain at the beginning of the follow-up interval; personality (neuroticism, extraversion); lifetime mental disorders (major depressive disorder, anxiety disorders [agoraphobia, panic disorder, generalized anxiety disorder, social phobia]); and intrapersonal correlations. The multinomial logistic regression models were fitted with no chronic pain at the end of the follow-up interval as the reference level.

Abbreviations: CP, chronic pain; RR, relative risk; 95CI, 95% confidence interval.

Bold entries mean significant results.

Regarding DN4 characteristics among participants with “multisite DN4+ CP,” 92.9% of them felt numbness, 85.7% presented sensations of tingling, 81.8% of pins and needles, 75% electric shocks, 72.7% of them felt burning, 37.5% had itching, and 25% had painful cold. Moreover, 60% of the participants with “multisite DN4+” CP reported sleep disorders (vs. 42.1% of “non-multisite DN4+,” 42.1% of “non-multisite DN4−” and 24% of those without CP, χ^2^ = 21.2, *p* < 0.0001), and 13.3% presented fatigue (vs. 23.8% of “non-multisite DN4+” group, 8.5% of “non-multisite DN4−” and 6.3% out of those without CP, χ^2^ = 25.5, *p* < 0.0001).

## Discussion

To our knowledge, this study is the first to assess the prospective associations of different types of ACEs with the incidence of CP over a 5.5-year period in a cohort of middle-aged and older community-dwellers. Our main findings were as follows: (1) the presence and the number of ACEs were linked to a higher risk of developing multisite CP over time; (2) among ACEs, parental divorce or separation was associated with higher incidence of multisite CP; (3) parental divorce or separation were strongly associated with multisite CP with DN4 characteristics as nociplastic ones; and (4) the higher CP occurrence in individuals with parental divorce or separation was not explained by potential confounders, including sociodemographic characteristics, comorbid mental disorders or psychological characteristics, antidepressant, or analgesic medications.

The strong association found between ACEs and multisite CP confirms findings from previous research where an excess of ACEs was associated with chronic multisite pain in numerous cross-sectional studies [16, 19, 20]. However, the majority of these studies have examined ACEs retrospectively in participants with or without multisite CP, and have been shown to be sensitive to recall bias: people remember past events with different degrees of accuracy, which can lead to inaccurate estimates of the risk associated with exposure [22]. Regarding longitudinal research, our results are compatible with the findings from Jones et al. [24] showing that family difficulties during childhood before 7 years were associated with widespread pain in adulthood, including a trend to significance for divorce/separation (OR 1.3, CI 0.99–1.7). However, in their study, childhood events were not directly measured from participants but by maternal report. According to the frequency of familial aggregation between widespread pain, in particular fibromyalgia, and familial depression, the authors suggested that the mothers of children who developed CP had higher rates of depression themselves, and could over-report ACEs in their children [24]. In our study, ACEs were collected from adult participants prior to the onset of CP, thereby excluding this bias.

Regarding the association between ACE types and multisite CP, our results differ from the literature insofar as parental divorce was the sole predictor of the development of multisite CP with neuropathic features. A recent large cross-sectional study showed that parental divorce was weakly associated with CP at midlife (OR 1.26), but not specifically with widespread pain [43]. On the other hand, we failed to show a relationship between sexual or physical abuse or loss of a family member, and the development of multisite CP, as frequently found in previous studies [12, 44]. In other respects, witnessing of violence between parents was significantly linked to multisite CP when assessed individually, but it became nonsignificant in model 2 when all types of ACEs were entered simultaneously. Indeed, witnessing of parental violence is frequent before and sometimes after divorce. However, in our cohort, parental divorce explained multisite CP independently from witnessing violence. Parental divorce is often accompanied by single-parent upbringing, usually by the mother. However, an earlier study highlighted the role of paternal education (care and encouragement of freedom) as a protective factor against CP in adulthood [45]. Moreover, raising children alone is probably more stressful for the single parent, and may reinforce neuroticism in children, and particularly high levels of neuroticism may positively influence the onset of CP [4]. High levels of neuroticism or extraversion may also modify the recall of ACEs, as may depression, favoring a negative bias in the recall of traumatic events. The association between parental divorce and multisite CP, particularly with neuropathic features, remained highly significant after accounting for these factors.

When considering our participants with multisite CP and DN4+, several symptoms were frequent, in particular, tingling, pins, and needles and sensations of numbness. These symptoms are also frequently found in fibromyalgia, which is known to be strongly favored by ACEs, and should prompt discussion of the differential diagnosis between neuropathic pain and fibromyalgia. Moreover, symptoms explored by the DN4 that are the most specific to neuropathic pain, such as the sensation of painful cold, were infrequent in our cohort. To clarify the type of pain in this group with multisite DN4+ CP, we assessed the prevalence of other core symptoms of fibromyalgia, notably sleep disorders, and chronic fatigue. The latter symptom was rarely present in our participants indicating that they were unlikely to be affected with fibromyalgia. Furthermore, as our participants had an average age of 58 years and fibromyalgia typically emerges before this age [46], we can assume that most participants with this condition would have already presented CP in the beginning of the first interval, and thereby, they would have been eliminated from the present analyses.

Previous research has highlighted that individual risks of developing CP may be increased by ACE-induced alterations during neurodevelopment in key neurobiological substrates, such as the hypothalamic-pituitary-adrenal axis [47, 48]. In addition, ACEs have been shown to modulate emotional responses to pain [4]. These effects may be the result of long-lasting ACE-related changes during early neurodevelopment. In CP patients, ACE-related alterations in intrinsic connectivity have been observed in the salience or executive control network, which is involved in central pain amplification. Connections between amygdala and prefrontal circuits have been shown to be particularly sensitive to early-life events [11]. Changes in these brain networks may contribute to the development of neural and behavioral patterns persisting during adulthood. In addition, frontal executive control becomes less effective with aging [49], which could explain the onset of CP in middle-aged and older adults [48].

### Limitations

Several limitations should be considered when interpreting our study results. First, adult memory of childhood trauma is subject to incomplete recall. However, due to the prospective design with information on ACEs being collected prior to the emergence of CP, the likelihood of reporting ACEs should not have been influenced by the outcome status, ruling out the risk of recall bias. Second, the DN4 questionnaire has only been validated in patients attending CP centers, where the prevalence of CP, particularly neuropathic pain, is high [29]. However, there are no validation data available from community samples, where the prevalence of neuropathic CP is much lower, varying from 7 to 10% [2, 50], which may decrease the accuracy of the DN4 questionnaire (its positive predictive value) for diagnosing neuropathic CP [51]. Furthermore, it would have been interesting to prospectively collect traumatic events and CP in childhood, and follow the development of CP in adulthood, but our cohort included subjects aged 35 and over. Indeed, as our analysis focused on subjects whose CP occurred after the age of 40, we were unable to take into account subjects whose CP occurred during childhood, adolescence, or early adulthood, which are known to be strongly associated with severe and/or multiple ACE-related trauma.

Moreover, exposure to at least one traumatic event during childhood was frequent in our study, but the prevalence of ACEs observed in our study is lower than that found in the literature [9, 52]. However, it is very difficult to compare the frequency of ACEs in the present study with that of other studies, as there are virtually no other studies that have evaluated exactly the same list of events. In a previous article, we compared the occurrence of well-specified PTSD events in our sample with that of other studies. Compared with studies carried out in most other European countries or outside Europe, the lower prevalence of lifetime exposure to traumatic events is consistent with that of two Swiss and German studies [53].

Additionally, PTSD could be a mediator between ACEs and CP. However, we found no association between sexual/physical abuse and witnessing domestic violence, which are typical triggers of PTSD. Nevertheless, a complementary analysis assessing the association between ACEs and CP also controlled for PTSD, which did not modify the results (data not shown). Finally, the limited number of subjects with both multisite CP+/DN4+ and a history of parental divorce may preclude the generalizability of the present results. Further longitudinal studies with a prospective collection of ACEs and CP, larger numbers of subjects, and longer FU are needed to confirm the present results.

## Conclusion

Our results show that among childhood adversities, parental divorce or separation, a frequent event that is considered quite trivial, strongly increases the risk of developing multisite CP as a middle-aged adult, specifically with neuropathic features, in a general population without CP at baseline. These results suggest the value of multimodal management, including psychotherapy, for the prevention or management of health problems including CP. Indeed, the clinical experience of CP centers suggests the usefulness of a multidimensional approach, integrating the patient’s autobiographical background. In particular, explaining to adults suffering from CP that their pain may be linked to events in their lives can help them to better understand and manage their pain.

## Data Availability

The data of CoLaus|PsyCoLaus study used in this article cannot be fully shared as they contain potentially sensitive personal information on participants. According to the Ethics Committee for Research of the Canton of Vaud, sharing these data would be a violation of the Swiss legislation with respect to privacy protection. However, coded individual-level data that do not allow researchers to identify participants are available upon request to researchers who meet the criteria for data sharing of the CoLaus|PsyCoLaus Datacenter (CHUV, Lausanne, Switzerland). Any researcher affiliated to a public or private research institution who complies with the CoLaus|PsyCoLaus standards can submit a research application to research.colaus@chuv.ch or research.psycolaus@chuv.ch. Proposals requiring baseline data only, will be evaluated by the baseline (local) Scientific Committee (SC) of the CoLaus and PsyCoLaus studies. Proposals requiring follow-up data will be evaluated by the follow-up (multicentric) SC of the CoLaus|PsyCoLaus cohort study. Detailed instructions for gaining access to the CoLaus|PsyCoLaus data used in this study are available at www.colaus-psycolaus.ch/professionals/how-to-collaborate/.
